# Current Status of Colorectal Cancer Screenings: Tailoring Them to Mississippi’s Rural Geography, Demographics, Infrastructure and Community Needs

**DOI:** 10.7759/cureus.97517

**Published:** 2025-11-22

**Authors:** Srinivasan Vijayakumar, Sudheer Koutha, Catherine Young, Vani Vijayakumar, Cecelia Brewington, Felisa Wilson-Simpson, Lilanta J Bradley

**Affiliations:** 1 Radiology, Ochsner Clinic Foundation and Ochsner Health, New Orleans, USA; 2 Department of Radiation Oncology, Manipal Comprehensive Cancer Care Centre Kasturba Medical College and Manipal Academy of Higher Education Manipal, Manipal, IND; 3 Cancer Care, Cancer Care Advisors and Consultants LLC, Ridgeland, USA; 4 Mississippi Comprehensive Cancer Control Program, Mississippi State Department of Health, Jackson, USA; 5 Office of Health Surveillance Research and Evaluation, Mississippi State Department of Health, Jackson, MS, USA; 6 Nuclear Medicine, Department of Radiology, Ochsner Medical Center, New Orleans, USA; 7 Radiology, Ochsner Medical Center, New Orleans, USA; 8 Office of Community Health Workers, Mississippi State Department of Health, Jackson, USA; 9 Community Medicine and Population Health, University of Alabama, Tuscaloosa, USA

**Keywords:** ai based modeling, colon capsule endoscopy, colonoscopy, colorectal cancer, colorectal cancer screening, ct colonography, deep south, fecal immunochemical test, global south, mississippi

## Abstract

Colorectal cancer (CRC) is a major cancer problem not only in western nations, but also now even in the developing world such as the Global South (GS). Within the US, the outcomes are worse in resource-scarce Deep South (DS) states including Mississippi (MS). The irony is that CRC can be diagnosed in precancerous and early stages with CRC screening (CRCS) - thus can stop progression to CRC (improving the survival outcomes). This irony is due to the low CRCS uptakes in MS and DS. Why CRCS uptake remains low in MS and DS was recently reviewed by us (“How to Change the Tide of Bad News to a Success Story”) and that the solution is in ‘using the right health care intervention at the right time for the right population’, using an interdisciplinary, continuum of care approach with an emphasis on the involvement of community health care workers (CHCW). Use of cutting-edge new innovations such as precision population medicine concepts that include telemedicine, wearable devices, socioeconomic deprivation indexes with an emphasis on community education including for the CHCW. However, the sheer number of CRCS options and possible combinations make it even more complex for an average practitioner (let alone for a CHCW) to comprehend and offer the right choice for the population at risk. To remedy, this second report in this series aims to serve as a comprehensive source describing various state-of-the-art options in CRCS as well as outlining the advantages and disadvantages of each. This ‘guide’ emphasizing an interdisciplinary approach as well as not using one-size-fits-all models in CRCS policies is likely to improve CRC uptake and outcomes in MS and DS. This team of interdisciplinary experts synthesized a conceptual framework from a peer-reviewed literature review of the past decade leading to new hypotheses, innovations and ideas for practice and future research. CRCS options recommended by professional societies, including invasive direct visualization (colonoscopy and sigmoidoscopy), non-invasive direct visualization (CT colonography and colon capsule endoscopy), and stool- and blood-based screening, are detailed, highlighting relative advantages, limitations, and optimal use scenarios. Using insights from simulation models and population-level studies, the cost-effectiveness and clinical outcomes of 13 different CRCS strategies are considered. Evidence from international randomized trials and national healthcare systems provided key perspectives on tailoring screening practices based on patient risk, access, provider readiness, and local infrastructure is presented. Cluster-randomized trials from rural U.S. regions supported the integration of CHCW and patient navigation in improving CRCS uptake are described. Finally, a conceptual framework to guide implementation of precision, community-tailored CRCS interventions in high-risk and underserved populations, aligning with current U.S. Multi-Society Task Force recommendations are proposed, focusing on equity, early initiation of screening, and informed choice of testing strategies. The paper concludes with listing short-, mid- and long-term potential practical implementation and research ideas. These steps, especially with the rapidly evolving technological and biological innovations, can lead to more successful, efficient and cost-effective CRCS strategies for the state of MS, other states in DS as well as similar communities in the Global South.

## Introduction and background

Colorectal cancer (CRC) is emerging as a major health care issue in the USA and the rest of the world [1]. CRC screening (CRCS) has been shown to pay great dividends by decreasing the incidence and mortality from CRC when successfully implemented [2]. However, there are major differences in the recommendations and practical/logistical implementation issues among nations and regions within nations. A good example is the state of Mississippi (MS) and the Deep South (DS) in the USA, which can serve as a model for the Global South (GS) with new approaches in CRCS. MS is leading the nation in CRC mortality during 2018-2022 with an age-adjusted mortality rate of 17.6 per 100,000 (37% higher than the national average of 12.9 per 100,000) [3,4]. The need for immediate attention to the CRC epidemic in MS is expanded in an accompanying paper [5]. Improvements in colorectal cancer survival have largely been driven by early detection initiatives and the removal of precancerous polyps through screening methods such as colonoscopy, flexible sigmoidoscopy, CT colonography, fecal immunochemical testing, and fecal occult blood testing [2]. In this report, currently recommended and optimal CRCS options in the USA as well as in other countries will be detailed. The rationale for various options and their effectiveness in decreasing the incidence of CRC as well as improving the overall survival for CRC will be described. The advantages and disadvantages of CRCS versus ‘No Screening’ will be outlined for an ‘average risk person.’ Given MS’s rural geography, high comorbidities, being among the DS states with many infrastructure constraints and its demography with poor socioeconomic metrics - recommendations for using the resources and options in a smart and cost-efficient way will be approached in an interdisciplinary manner. This approach and its success can serve as a pilot demonstration model for DS and GS. Although this report focuses on resource-lean states and provinces such as Mississippi in the USA, the comprehensive synthesis of data and approaches, we believe it can be useful to anyone interested in the status of CRCS in circa 2025.

## Review

Methodology

An initial PubMed search was performed by a clinical oncologist with an added public health background. Only English language literature was searched. The key words used were colorectal cancer, rectal cancer, colorectal cancer screening, colonoscopy screening, flexible sigmoidoscopy, sigmoidoscopy screening and Mississippi. The focus was on the peer-reviewed papers published in the past 10 years. First the abstracts were reviewed, and any relevant papers were then reviewed in full. Further searches in PubMed, Google Scholar and Google were carried out using a combination of index terms listed above and ‘similar papers’ that were yielded from those searches. Finally, additional search terms were used, for example, Cologuard, Cologuard plus, etc. The flowchart in Appendix 1 details our approach to an extensive search of relevant peer-reviewed papers in the past 10 years. This paper cannot be considered as an extensive review following the criteria of Preferred Reporting Items for Systematic Reviews and Meta-Analyses (PRISMA) and thus may have biases.

However, these potential biases were avoided by the following steps. In the next step a review of the initial searches made and drafts written by the initial clinical oncologist were conducted by an epidemiologist, a community health and population medicine researcher and a public health policy leader. In the next step, a community health worker leader reviewed the drafts. Finally, two practicing academicians with radiology, disparities research and internal medicine backgrounds reviewed the final three drafts. Using in-person discussions, video and telephone conferencing, and email sharing of drafts, additional conceptual constructs, hypotheses generation, additional literature searches and modifications were made. The final draft was read and approved by all the contributors. All investigators contributed to different conceptual constructs of the paper and hypothesis, literature searches and reviews and writing of the paper.

Statement of the issues in CRCs related to the state of Mississippi

It is well documented that CRCS helps detect colonic mucosal lesions (variously called adenomas and/or polyps) that have the potential to lead to a malignant tumor many years (one to 15 years) ahead of time [6]. They tend to bleed, but not always. If they bleed even minimally, they can be detected by testing for occult blood in the stool. If they do not bleed, such tests will miss them. On the other hand, tests that can directly visualize them, such as a colonoscopy and CT colonoscopy (for the entire colorectum) or a sigmoidoscopy (for the distal colon and rectum), can detect the lesions (including the non-bleeding ones). The direct visualization tests therefore have higher sensitivity for detection of precursor cancerous polyps. Prevention is possible with direct visualization tests as they can lead to polypectomy with colonoscopy. However, colonoscopy and sigmoidoscopy are invasive procedures that need (a) bowel preparation, (b) expertise (experienced physicians), (c) more infrastructure (and thus are more costly), (d) a break from the person’s work schedule (thus leading to increased reluctance to undergo such procedures as a first step), (e) anesthesia and recovery and (f) consideration for potential (although minimal) complications such as bleeding and/or perforation of the bowel [6-10]. As noted more expansively in a parallel publication, CRC is a major cancer issue in MS as well as in the DS [5].

We hypothesize the following: (a) New innovations in CRCS, precision population medicine (PPM) and information communication technology (ICT) can be optimally customized to local communities’ needs to improve the CRCS acceptance, uptake and utilization [11], (b) This will need to take advantage of the already existent resources of the community health workers (CHW) in MS for efficient and cost-effective implementation of risk-adjusted and person-specific CRCS options, (c) These efforts if and when implemented with an interdisciplinary team can lead to a reasonably rapid achievement of nationally recommended CRCS uptake targets and thus can improve CRC outcomes in MS, (d) This success can serve as a pilot model for other states in the DS as well as other regions and countries sharing similar rural, socio-economic, infrastructure and a paucity of health professionals availability issues for improving cancer care not only in CRC but also in other cancers such as cancer of the uterine cervix - a cancer with many unique characteristics that can potentially be eliminated [12,13].

In the subsequent sections, various CRCS options, their advantages and disadvantages, their effectiveness reported in clinical and population-based studies and hurdles for practical implementations and logistical ‘roadblocks’ will be described. How the evolving new CRCS options such as ‘liquid biopsies' and risk-adjusted CRCS approaches can help a state like MS will be expanded. Together with the companion paper, one hopes that a robust roadmap to improve CRCS and CRC outcomes in MS, DS and GS evolves from these interdisciplinary reviews and perspectives.

CRCS recommendations from professional societies and an overview of CRCS options

There are many effective options for CRCS [7]. In this section, the focus will be on ‘average risk’ and the asymptomatic population. Although there are many professional societies with slightly differing recommendations for CRCS - both in terms of age criteria as well as the preferred first and/or subsequent choices of testing, there seems to be a congruence/consensus overall. A summary of the professional societies’ recommendations is shown in Table 1. The nuanced differences among the recommendations for disadvantaged populations, especially related to Mississippians, MS’s demographics, rural geography, and infrastructure limitations, have to be carefully considered to overcome the overwhelming CRC issues in the state of MS, and these will be expanded later. This report in general focuses on ‘average risk’ population. An ‘average risk’ person is generally defined as someone without (a) A personal history of colorectal cancer or certain types of high-risk polyps, (b) A family history of colorectal cancer, (c) A personal history of inflammatory bowel disease (ulcerative colitis or Crohn’s disease), (d) A confirmed or suspected hereditary colorectal cancer syndrome, such as familial adenomatous polyposis (FAP) or Lynch syndrome (hereditary non-polyposis colon cancer or HNPCC), (e) A personal history of getting radiation to the abdomen or pelvic area to treat prior cancer (Table 1).

**Table 1:** Colorectal cancer screening (CRCS) recommendations of professional societies in the USA Sources: [7,9,10,15] Definition of Average Risk from American Cancer Society: *For screening, people are considered to be at average risk if they do not have: A personal history of colorectal cancer or certain types of polyps A family history of colorectal cancer A personal history of inflammatory bowel disease (ulcerative colitis or Crohn’s disease) A confirmed or suspected hereditary colorectal cancer syndrome, such as familial adenomatous polyposis (FAP) or Lynch syndrome (hereditary non-polyposis colon cancer or HNPCC) A personal history of getting radiation to the abdomen (belly) or pelvic area to treat a prior cancer This table is independently developed by authors from the sources cited.

Professional Society / Agency	Age Criteria for Average Risk Adults	Age Criteria for the ‘Elderly’ And Other Specifics
US Preventive Services Task Force (USPSTF) [7]	45 – 75 YEARS Recommended screening strategies include the following:	Selective screening 76 – 85 years (considering the patient’s overall health including comorbidities, prior screening history, and patient’s preferences).
	High-sensitivity guaiac fecal occult blood test (HSgFOBT) or fecal immunochemical test (FIT) every year	
	Stool DNA-FIT every 1 to 3 years	
	Computed tomography colonography every 5 years	
	Flexible sigmoidoscopy every 5 years	
	Flexible sigmoidoscopy every 10 years + annual FIT	
	Colonoscopy screening every 10 years	
American Cancer Society (ACS) [15]	Starts at age 45 years and continue until 75 as long as in good health with a life expectancy of 10 years. Recommended Test Options are listed below:	76-85 years; to be decided on an individual’s preferences, life expectancy, overall health, and prior screening history.
	Highly sensitive FIT every year	
	Highly sensitive guaiac-based fecal occult blood test (Gfobt) every year	
	Multi-targeted stool DNA test with fecal immunochemical testing (MT-Sdna or Sdna-FIT or FIT-DNA) every 3 years	
	Colonoscopy every 10 years	
	CT colonography (virtual colonoscopy) every 5 years	
	Sigmoidoscopy every 5 years	
	NOTE: If a person chooses to be screened with a test other than colonoscopy, any abnormal test result should be followed up with a timely colonoscopy	
US Multi-Society Task Force on Colorectal Cancer (USMSTFCRC) [9,10]	Starting age is 45 and in congruence with other professional societies (ACS, and USPSTF) in terms of other age criteria	People without prior history of CRCS in good health and life expectancy of 10 or more years can be offered CRCS
	Stopping age is 75 among those with consistent previous screening history with negative test findings, especially a high-quality colonoscopy	
	Stop at an earlier age if life expectancy decreases to less than 10 years	
	Tests included are: colonoscopy, FIT, FIT-fecal DNA, CT Colonography, flexible sigmoidoscopy, and capsule colonoscopy	
	A multiple options discussion approach versus sequential offerings versus risk-stratified approach are all acceptable based on circumstances	

The starting age of CRCS has been brought down to 45 years from 50 and Table 1 confirms a general agreement among different professional societies on this. However, there are a few differences between CRCS guidelines after the age of 70-75. It is safe to state that if the expected life expectancy is 10 or more years, a CRCS can be discussed with a person with an average risk. In general, these guidelines can be followed in MS. The unknown aspect that needs to be addressed is the higher risk of CRC among African Americans and whether there should be a lower age to start screening, like prostate cancer being considered by some [14]. Since almost 40% of MS’s population is African American, this is an important question that needs to be answered in the future.

Table 2 describes the currently available tests and procedures for CRCS [3]. Currently stool-based fecal immunochemical test (FIT) is the most common one used worldwide, looking for occult human hemoglobin. Hence, these stool-based tests are also called fecal occult tests (FOT). FIT has many advantages over the older version of the ‘guaiac’ stool test. Although both tests detect occult hemoglobin, FIT is more sensitive and specific, and there are other advantages, as detailed in Table 3. In randomized studies, stool-based (at the time of these studies, either FIT or fecal guaiac-based test (FGBT)) CRCS versus colonoscopy have been compared to see what the preferred option is chosen by the screening-eligible populations. These studies compare FOT versus colonoscopy as the two arms of a study or in a sequential manner where FOT is used as a ‘first step’ occult blood detection tool in asymptomatic eligible populations, followed by ‘direct visualization’ methods (Tables 1-7). Invariably, FOT is a preferred choice due to its noninvasive approach as well as the convenience of getting samples done at home or in a clinic without interruption to daily activities. FGBT is almost seldom used in the USA anymore and as detailed in Table 3, it should be avoided. Currently FIT is the preferred FOT in the USA (Table 3). A potentially improved FOT is the fecal multi-target DNA test (FMTDNAT). The next section will detail the status of FMTDNAT in the USA.

**Table 2:** Currently Available Tests and Procedures for Colorectal Cancer Screening (CRCS) Sources: [7,16-32] Table has been independently developed by the authors from the sources cited

General Category of the Tests	Specific Tests Within Each Broad Category	Comments	References
Stool-Based Tests	Fecal Immunochemical Test (FIT) A type of Fecal Occult Test (FOT) detecting human hemoglobin molecules	Most common test worldwide; Class I evidence showing survival [colorectal specific] improvements exist	Shaukat and Levin, 2022 [7]; Chiu et al. 2015 [16]; Logan et al. 2012 [17]; Shaukat et al. 2013 [18]
	Fecal Guaiac-Based Test (FGBT) An older version of FOT again detecting human hemoglobin	Refer to Table 3 for expanded details of FGBT and the pros and cons versus FIT	Refer to Table 3
	Fecal multi-Target DNA test (FMTDNAT)	Potential cost-effective screening test for the resource-constrained populations	Pickhardt PJ 2016 [19] Imperiale TF et al. 2014 [20] Imperiale TF et al. 2024 [21] Carethers JM 2024 [22]
Direct Visualization: ‘Invasive’ or ‘Interventional’ Procedures	Flexible Sigmoidoscopy (FS)	Advantage: Distal Colon Visualization (DCV) and Lesion Removal Capability; High pre-cancerous polyp detection sensitivity; Pathology can be ascertained	Gangwani et al. 2023 [23]; Atkin et al. 2010 [24]; Wooldrage et al. 2024 [25]; Wang et al. 2023 [26]
	Colonoscopy	Advantage: Total Colon Visualization (TCV) and Lesion Removal Capability; Pathology can be ascertained	Refer to Table 12
Direct Visualization: Non-Invasive or Non-Interventional Procedures	CTC (Computerized Tomographic Colonography)	Advantage: TCV & No anesthesia; High pre-cancerous polyp detection sensitivity.	N/A
		Disadvantage: If a lesion is found, it will need colonoscopy or FS for removal and pathology to ascertain	
	Colon Capsule Endoscopy (CCE)	Advantage: TCV	N/A
		Disadvantage: If a lesion is found, it will need colonoscopy or FS for removal and pathology to ascertain.	
Blood Tests	FDA Approved	Epi proColon 2.0 was approved in 2016. However, CMS did not approve for reimbursement, and the production has been discontinued	[27-32] Lamb YN et al. 2017 [28]; Fillon M 2024 [29]; Kandel A et al. 2025 [30]; Patelli G et al. 2025 [31]; Van den Puttelar R et al. 2025 [32]
		Guardant Health Shield Test was approved in 2024 and is also CMS approved	

**Table 3:** Comparison of Fecal Immunochemical Test (FIT) Versus Fecal Guaiac Based Test (FGBT) Authors’ Recommendations: FGBT needs to be abandoned in favor of FIT or other newer tests (expanded later in this paper), as many cancer care organizations are recommending the use of FIT exclusively for fecal occult tests (FOT) [35,36] Use FIT more than once: Multiple testing with FIT can make FIT more comparable to advanced colorectal cancer (CRC) detection compared to colonoscopy. Annual or biennial use of FIT has the following advantages compared to colonoscopy: Better acceptance than colonoscopy; A better safety profile; Ease of testing; Cost utility – although the initial cost of FIT may be slightly higher than FGBT, the ultimate cost of detecting a CRC is less due to FIT’s higher sensitivity/lower false positive rates, and decreasing unnecessary colonoscopies; FIT also improves the quality-adjusted life years (QALY) [36,37] Attention should be paid to FIT quantification thresholds in the use of FIT-based CRC screening (CRCS). The flexibility of quantitative FIT and being able to use numerical values are added advantages of FIT. This can help customization for each CRCS program/community/clinical trial [36-38]. Sources: [7,33-38] The table has been independently developed by authors from the sources cited.

ITEMS	FIT	FGBT	Comment
Molecule detected	Hemoglobin	Hemoglobin	N/A
Chemical technique	Antibodies specific to human hemoglobin	Nonspecific chemical [peroxidase reduction] reaction	N/A
Number of standard samples required	Usually, One	Usually, Three	N/A
Sensitivity and specificity	Higher	Lower	N/A
Influence of diet and medications on results	None	Present	Thus, requiring modifications of diet and medications before testing
Uptake (Successful Completion of screening for CRC)	Higher	Lower	van Rossum et al. 2008 [33]; Shaukat and Levin, 2022; [7] Akram et al. 2017 [34]
‘Number to (Colono-)Scope’ after successful testing	Equal		van Rossum et al. 2008 [33]
Detection of advanced polys and/or malignancies	Higher	Lower	van Rossum et al. 2008 [33]; Shaukat and Levin, 2022 [7]
Ability for quantitative measurements of occult fecal hemoglobin concentration ((f-Hb) measured in µg hemoglobin/g feces)	Possible	Not possible	This gives advantages to FIT since these values can be customized to each program’s infrastructures and funding abilities, as well as enablement of detailed data analysis in clinical trials

**Table 4:** Modified Summary from Cochrane Database Systematic Review The table has been reproduced as is from reference [39] with permissions. FIT = fecal immunochemical test, FGBT = fecal guaiac-based test, CRC = Colorectal cancer

Items	Details	Comments	
Number of studies analyzed	63 studies	Large study	
Number of individuals included	Close to 4 million	Good statistical validity expected	
Theoretical extrapolation 1	If 10,000 people take part in screening with a fecal blood test and 100 people in this group have CRC, 24 will be missed if FITs are used for screening, versus 61 will be missed using FGBT	FIT is a better test in terms of specificity than FGBT	
Theoretical extrapolation 2	If 10,000 people take part in screening with a fecal blood test and 1000 people in this group have large polys or CRC or both, 670 will be missed if FITs are used for screening versus 850 will be missed using FGBT	FIT has better sensitivity	
Theoretical extrapolation 3	If 10,000 people take part in screening with a fecal blood test, 594 in each group will undergo unnecessary colonoscopy (despite not having a CRC) due to false positive test results	FIT misses fewer CRCs than FGBT, however, an equal number of people will undergo an unnecessary colonoscopy	
Reliability of the 63 studies	The studies are reasonably reliable since they met the quality criteria specified by Cochrane’s Systematic Review before commencing the analysis.	The Cochrane Database Systematic Review [35] results can be used to make conclusions that can be practice changing	

**Table 5:** Salient Findings of Blue-C Clinicaltrials. Gov Nct04144738 NG-FMTDNAT Study The table is adapted and modified from sources [20,21] FMTDNAT = fecal multi-target DNA test, NG = Next Generation

NG-FMTDNAT [Cologuard Plus] Results (2024)	FMTDNAT [Cologuard] Results (2014)	
There were 20,176 participants – normal risk adults over 40 years of age undergoing colonoscopy.	9989 participants, 50 years or older and limited to 84 years and asymptomatic adults.	
98 (0.5%) had colorectal cancer.	65 (0.7%) had colorectal cancer.	
2144 (10.6%) had advanced precancerous lesions.	757 (7.6%) advanced. precancerous lesions (advanced adenomas or sessile serrated polyps measuring ≥1 cm in the greatest dimension’) on colonoscopy.	
6973 had non-advanced adenomas	6274 had negative findings	
10,961 had nonneoplastic findings or a negative colonoscopy.	Sensitivity for detecting colorectal cancer was 92.3% with DNA testing and 73.8% with FIT (P=0.002).	
Sensitivity for colorectal cancer was 93.9% (95% confidence interval [CI], 87.1 to 97.7).	Specificity was 86.6%.	
Specificity for advanced neoplasia was 90.6% (95% CI, 90.1 to 91.0).	Specificity for negative findings was 86.6%.	
Sensitivity for advanced precancerous lesions was 43.4% (95% CI, 41.3 to 45.6.	The sensitivity for detecting advanced precancerous lesions was 42.4%.	
Specificity for non-neoplastic findings or negative colonoscopy was 92.7% (95% CI, 92.2 to 93.1).	The rate of detection of polyps with high-grade dysplasia was 69.2%	

**Table 6:** Summary of Comparison Between Next-Generation Improved Multi-Target DNA Test Versus Older Version of the Multi-Target DNA Test The table is adapted and modified from sources [20,21] CRC = Colorectal cancer

Item	Findings	Comments
Main shortcoming	The newer and older versions of the Multi-Target DNA Test were not compared in a head-to-head manner.	This must be carefully considered in clinical decision makings and cost-Benefit comparisons
Sensitivity for CRC detection of both tests	Comparable	About 90+%
Sensitivity for advanced precancerous lesions	Low, but comparable	About 49+%. The low sensitivity of the newer version of the test is disappointing
Specificity for non-neoplastic / negative colonoscopy findings	improved from about 87% for the older version of the test to 92% for the newer version of the test	The 5% improvement is impressive, if real, since one must be cautious in drawing firm conclusions given that this observation is an extrapolation and not from a head-to-head comparison

**Table 7:** Fecal Immunochemical Test (FIT) Results Compared With Cologuard Plus or Cologuard From Two Different Studies The table is adapted and modified from sources [20,21]

FIT vs. Cologuard Plus Results	FIT vs. Cologuard Results
Sensitivity for CRC was 67.3% (FIT) (95% CI, 57.1 to 76.5) vs. 93.9% (95% confidence interval (CI), 87.1 to 97.7) for next generation testing (NGT) (P<0.001)	Sensitivity for colorectal cancer (CRC) was 73.8% with FIT (vs. 92.3% for DNA testing) (P=0.002).
Sensitivity was 23.3% (FIT) (95% CI, 21.5 to 25.2) vs. 43.4% (NGT) (95% CI, 41.3 to 45.6), for advanced precancerous lesions. (P<0.001)	Sensitivity for detecting advanced precancerous lesions was 23.8% vs. (42.4% with DNA testing) (P<0.001), detection rate of polyps with high-grade dysplasia was 46.2% with FIT vs. (69.2% with DNA testing) (P=0.004), detection rate of serrated sessile polyps measuring 1 cm or more was 5.1% vs. (42.4% for DNA testing) (P<0.001).
Specificity for advanced neoplasia was 94.8% (FIT) (95% CI, 94.4 to 95.1) vs. 90.6% (NGT) (95% CI, 90.1 to 91.0) (P<0.001).	Specificities were 94.9% with FIT vs. 86.6% (DNA testing) among participants with non-advanced or negative findings (P<0.001)
Specificity for non-neoplastic findings or negative colonoscopy was (FIT) 95.7% (95% CI, 95.3 to 96.1) vs. (NGT) 92.7% (95% CI, 92.2 to 93.1).	Specificities were 96.4% (FIT) vs. 89.8% (DNA), respectively, among those with negative results on colonoscopy (P<0.001).

Fecal multi-target DNA tests: Cologuard versus Cologuard plus

A new stool-based test is FMTDNAT, which was approved by the FDA in August 2014. It is made by Exact Sciences (Madison, WI, USA) [40] and is named Cologuard™. Center for Medicare and Medicaid Services (CMS) approved FMTDNAT-Cologuard for Medicare Part B reimbursement in October 2014 [41]. FMTDNAT-Cologuard is available only in the USA currently [42]. In 2014, the approval criteria for FMTDNAT-Cologuard included (a) age 50 or older adults with (b) an average risk for CRC. FMTDNAT-Cologuard was designed to detect human hemoglobin (i.e., like FIT) plus detection of mutations associated with CRC in the DNA of cells shed by advanced adenomas or CRC (quantitative molecular assays for KRAS mutations, aberrant NDRG4 and BMP3 methylation, and β-actin, plus a hemoglobin immunoassay) [7,20]. The stool sample can be collected conveniently at home, although there are specific details involved [19]. These steps include (a) a collection kit being sent home, (b) defecation into a plastic container, (c) a manual agitation of the stool specimen using a handheld probe, (d) adding a preservative over the stool sample, (e) preparing the entire sample for overnight shipping to the central laboratory of Exact Sciences [19]. In the laboratory, the specimen is tested for seven DNA mutation biomarkers, two DNA methylation biomarkers, and β-actin as a control for human DNA. In addition, human hemoglobin is also tested as a FIT. Currently, quantitative analysis results are not reported, and qualitative positive/negative results are sent. This study is also called ‘Deep-P Study’, implying ‘Deep Phenotype Study’.

In October 2024, an improved version of Cologuard™ named Cologuard Plus™ was approved by the FDA [43]. Cologuard Plus™ is a next-generation multi-target DNA FOT. A prospective study results reported by Imperiale et al. 2024 [21] included asymptomatic adults 40 years of age or older, all of whom were undergoing colonoscopy for CRCS. All participants were initially tested with Next-Generation-FMTDNAT (NG-FMTDNAT (Cologuard Plus™)) and a commercially available FIT. The end points of the study were: (a) sensitivity of the tests for colorectal cancer, (b) specificity for advanced neoplasia, meaning CRC and/or advanced precancerous lesions, (c) specificity for nonneoplastic findings or negative colonoscopy, (d) sensitivity for advanced precancerous lesions and (e) comparison of NG-FMTDNAT with FIT. Advanced precancerous lesions were defined as identification of adenomas or sessile serrated lesions measuring 1 cm or more in the longest dimension, lesions with villous histologic features, and high-grade dysplasia [21]. Table 5 summarizes the results and compares the outcomes with the original Cologuard study findings of 2014 [20].

It is important to note that in the 2024 publication of the Cologuard Plus study [21], there was no head-to-head comparison of the use of Cologuard vs. Cologuard Plus. There was only a head-to-head comparison of contemporaneous FIT vs. Cologuard Plus findings. So, the comparisons outlined in Table 5 between Cologuard vs. Cologuard Plus are from two different populations with slightly different age group selection criteria, with almost 10 years between the two studies. So, the findings must be interpreted cautiously.

John Carethers [22], in an editorial that accompanied the Cologuard Plus results’ publication in 2024, made many important observations and some of them, including other considerations by the authors are expanded below: (a) The Deep-P study was designed to improve on the previous FGBT and FIT results showing detection of earlier-stage cancers, thus allowing an improvement in potential cure rates. In large, randomized studies, even FGBT CRCS, a less sensitive test than FIT, led to a reduction in mortality from CRC. (b) With the use of multiple DNA mutational targeting in the Deep-P study, sensitivity for the detection of colorectal cancer improved (Table 6). The sensitivity for detecting advanced adenomas was reasonable, although it needed further improvement. Unfortunately, the specificity of FMTDNAT was lower than FIT (Table 7). This can lead to unnecessary additional colonoscopies, thus adding potentially more complications, poorer Quality of Life (QOL), and overall cost. (c) The goal of achieving an 80% screening rate for general, average-risk populations in the USA is lagging by about 10% and even more among disadvantaged populations, one of the reasons being reluctance to undergo an invasive procedure (colonoscopy) as the first test. A non-invasive test with higher sensitivity and specificity can lead to higher uptake rates, thus potentially helping to save lives with early curable detection of CRC [22].

NG-FMTDNAT is a good example of such a strategy; however, the current limitations of NG-FMTDNAT are outlined in Tables 8, 9, including uncertainty about the lack of head-to-head clinical trials of FIT vs. the most recent state-of-the-art NG-FMTDNAT (Tables 5-10). Currently, the consensus regarding the FOT appears to be that all three available FDA-approved tests - FIT, FMTDNAT, and NG-FMTDNAT - as equally good options with some nuanced sensitivity, specificity, and practical user-friendliness differences. Table 8 compares over the counter (OTC)-FIT versus prescription-based NG-FMTDNAT. The recommendation that fecal-DNA-based tests can be repeated once in three years among those tested ‘negative’ versus yearly for FIT can make the former help improve the uptake and compliance as a non-invasive test of choice. However, the need for a prescription for fecal-DNA-based tests versus FIT (OTC available) can argue in favor of FIT. The ability to adjust the quantitative contents of occult hemoglobin with FIT versus the proprietary nature of the algorithm used for fecal-DNA-based tests, and the almost 20 times more expensive nature of fecal-DNA-based tests versus FIT are the other considerations in choosing the ‘correct’ FOT for a given circumstance.

**Table 8:** Fecal Immunochemical Test (FIT) Versus State-of-the-Art Next-Generation Fecal Multi-target DNA Test (NG-FMTDNAT) Compared – Current Status and Future Prospects References: [7,43-46]; the table has been developed by authors independently from the cited sources. QALY = quality-adjusted life years, CMS = Center for Medicare and Medicaid Services, FGBT = fecal guaiac-based test, CRCS = colorectal cancer screening

What is Being Compared?	FIT	State of the Art NG-FMTDNAT	Comment
What is detected in the stool?	Fecal human hemoglobin.	Fecal human hemoglobin plus methylated DNA markers; aberrantly methylated NDRG4 and BMP3; any of seven KRAS point mutations.	Current FDA-approved cut-off for FIT is 20 μg of hemoglobin per 1 g of stool
Cost if there is no health insurance coverage?	About $25	About $600	The cost-effectiveness comparison when QALY is the endpoint remains controversial at this time.
What is the likely cost for out-of-pocket payment?	Approximately $25.	Approximately $600.	Generally covered by CMS and most private medical insurance carriers in the USA.
Can they be purchased over the counter (OTC) in the USA?	Yes (as well as FGBT)	No. Requires a prescription from the Provider.	N/A
Is there an FDA ‘cut off’ for fecal hemoglobin content?	20 μg/g of hemoglobin	No. The currently approved FDA test is likely to be positive if the fecal hemoglobin goes above 100 ng/mL	These differences may explain the sensitivity differences between the two tests for advanced precancers.
Is quantitative adjustment of Fecal Hemoglobin possible?	Yes	Uses a proprietary mathematical algorithm for test positivity determination.	This is a current advantage of FIT in CRCS ‘customized’ programs; for example, using a threshold of 10 μg/g increases sensitivity to 91%, however, reducing specificity to 90%.
What is the recommended frequency?	Annual	Every three years.	Compliance is likely to be higher if the frequency of testing is lower.

**Table 9:** Future Research Recommendations From US Multi-society Task Force on Colorectal Cancer (USMSTFCRC) and How the Evolving ‘New Breakthroughs’ Can Shape Colorectal Cancer Screening (CRCS) Approaches in Mississippi (MS) Left column is reproduced from [9] with permission; right column is created by the authors.

Areas	Recommendations From US Multi-society Task Force on Colorectal Cancer (USMSTFCRC)	Potential Research Questions and Practical Applications to Mississippians
Patient selection	Starting age: Should age to start be the same for general population or determined by precision screening?	What should be the starting age for African Americans with known higher incidence of CRC, even if they otherwise belong to so-called ‘normal risk’?
	Stopping age: Relative impact of age, prior screening history, CRC risk, patient preference, and comorbidities.	Given a higher incidence of obesity, diabetes and other co-morbidities among Mississippians, what adjustments need to be made?
Provider acceptance	Provider attitudes and behaviors regarding starting screening earlier, test selection, and stopping screening.	Given the paucity of sufficient providers and colonoscopy capacity due to Mississippi’s rural nature, what are the solutions?
Screening test selection	Menu of equivalent options vs tiered approach vs hybrid approach	What will work for Mississippians? Does these options be tailored further based on other factors within MS?
Access, equity, compliance	Track disparities in access to and use of screening tests, diagnostic tests, and treatment interventions to address screening underuse in medically underserved populations	All the three items on the left – access, equity and compliance – fully apply to the state of MS.
Primary prevention	Populations that benefit from chemoprevention Optimal dietary and lifestyle recommendations	Mississippians need answers to the same question on the left, even more than the overall US population based on SDI and SDeI.

**Table 10:** Currently Preferred Colorectal Cancer Screening (CRCS) Methods in Different Countries CRC = colorectal cancer; EU = the European Union; FIT = fecal immunochemical test; hsFOBT = high sensitivity fecal occult blood test * NOTE: FIT appears to be the most preferred CRCS test in nine of 11 countries listed [7]. This is likely due to the convenience of testing, acceptability and cost-effectiveness. Adapted with permission from cited sources with added modifications.

Country/ Region	Age to initiate screening (years)	Age to stop screening (years)	Modality and Frequency
Canada (Variations exist across provinces)	50	74	Biennial FIT and hsFOBT
EU	50 (55 or 60 in some regions)	74	Biennial FIT (although Germany uses colonoscopy at 55)
UK	50	74	Biennial FIT
Pan American Region	50	74	Biennial FIT
USA	45	85	Multiple options, colonoscopy is most commonly used
South Korea	50 and older	Not Specified	Annual FIT
Australia	55	75	Biennial FIT
Japan	40	No Upper limit	Annual FIT
Taiwan	55	75	Biennial FIT
Israel	50	74	Annual FIT
Abu Dhabi	40	Not specified	Colonoscopy

Conclusions From NG-FMTDNAT/FMTDNAT Versus FIT Testing

It is not clear regarding the reasons for a decreased sensitivity in detecting CRC with the new study (FIT) where the sensitivity for CRC is 67.3% versus 73.8% in the older study. Otherwise, the FIT results from the two studies are comparable, as well as the other differences found between FIT vs. NG-DNA-Test. The approved once-in-three-years testing frequency of the NG-DNA test can help improve compliance among the populations at risk.

The options to be offered to Mississippians must take into account many factors outlined in the parallel paper that is focused on the current dismal CRCS status in the state of MS and how to improve the CRCS uptake and decrease the unnecessary deaths from CRS in the state of MS [5]. These considerations are further expanded in Table 9. The US Multi-society Task Force on Colorectal Cancer (USMSTFCRC) is a multispecialty task force with experts from three important gastrointestinal diseases-related professional societies. American College of Gastroenterology, the American Gastroenterological Association, and the American Society for Gastrointestinal Endoscopy are members of USMSTFCRC. USMSTFCRC's latest CRCS guidelines were published in 2017, followed by an update in 2022 [9,10]. This task force’s future research recommendations are shown in the left column in Table 9 and in the right column, the relevant questions related to MS are asked. For example, the guidelines for African Americans in terms of age at the initiation of the first CRCS. Even in its 2017 guidelines, USMSTFCRC recommended starting age at 45 years for African Americans. There needs to be further research in terms of other aspects specific to MS as listed in Table 9. Some of the recommendations espoused by Jones et al. [47] in the early part of 2025, specifically to improving cancer care in MS, need to be carefully considered in relation to CRCS also.

Before other CRCS options such as ‘direct visualization tests’ listed in Table 2 are taken up, it needs to be pointed out that FOT - mostly FIT - is preferred in most nations having published guidelines on CRCS (Table 10). Except Abu Dhabi and Germany (Table 10, under EU) other nations prefer FOT to direct visualization procedures. In the USA, multiple options (Table 2) are available, and multiple strategies are practiced (Table 11). The preference shown for FOT around the world is based on its ease of testing, potential for home-based testing, more eagerness of acceptance from the populations at risk, associated lower costs since expensive infrastructure (such as those needed for fibro optic sigmoidoscopy (FOS) and/or colonoscopy), avoidance of break in work schedule since anesthesia required for FOS or colonoscopy are not required and finally, easy accessibility to FOT even in rural areas. The advantages and disadvantages of these approaches will be discussed in the subsequent sections. Suffice to say that the accepted standard of practice in the USA is to consider many options outlined in Tables 1, 2. However, how these options are offered and/or presented to a person at risk for CRC widely differs, and there is no uniformity in the approach (Table 11) [48].

**Table 11:** Cost-Effectiveness Findings of Comparing 13 Different Colorectal Cancer Screening (CRCS) Strategies Using Marcov Plus Microsimulation Screening Analysis (MISCAN) models Table has been created by the authors from source [48]

ITEM	FINDINGS	COMMENTS
Take home message	CRCS was associated with a 5% to 23% relative-risk reduction and a 12% to 34% cancer-specific mortality risk reduction versus no screening	These findings are the basis of the reason the high mortality of CRC is considered potentially preventable
The best risk-reduction strategy	Use of colonoscopy, with the lowest incidence of CRC	These findings persisted, including when colonoscopy was included in all positive tests after the other screening tests
Results with the inclusion of ‘adenoma detection’ in the modeling of DNA testing strategies	Led to an improvement in their effectiveness – the life-years gained and the number of prevented cancers increased.	This finding is an attestation of the reason why CRC is considered ‘preventable’ (with the diagnosis and treatment of precancerous adenomas)
Cost-effectiveness of different strategies	CRCS strategies, in most cases, had costs that were significantly lower than the costs of treatment for CRC detected when no screening was offered. In addition, colonoscopy was the most effective strategy at the lowest cost	Any CRCS test is beter than none. However, currently colonoscopy is the best options (if patient can tolerate it without high risk of potential (predicted) complications)
Relative cost of different tests	Fecal DNA testing was more expensive than other strategies until the cost of fecal DNA testing was brought down to $29, and adenoma detection was also included in the model.	The cost of fecal DNA testing is likely to keep decreasing with new technology in the future. Yet, in resource-scarce populations and nations, the current cost-considerations are important in national health policy making

Strategies in terms of what options need to be presented and offered to an individual person, in what sequences, and how many options at a given time depend on many factors [10]. These include (a) if the encounter with the individual is in a dedicated CRCS setting or (b) during an opportunistic clinical encounter. The other factors are (c) infrastructure availability, (d) the background, training, experience, and comfort level of the professional offering the test, (e) urban versus rural setting (if multiple encounters and discussions are needed), and (f) cost, affordability, and insurance coverage considerations.

Under opportunistic settings, three optional strategies exist [10]: (a) Multiple options are presented describing pros and cons of each, including cost and inconveniences involved. (b) A sequential approach is executed. The professional’s first choice, consistent with Tables 1-4, is offered, followed by others if the patient declines the first, second, etc. (c) A risk-stratified approach is used. In high-risk situations, colonoscopy is offered first, followed by other options, versus in the reverse order among those considered to have a ‘lower risk’ of CRC development. The future research questions raised by Patel et al. [9] and the added importance of those research initiatives to the state of MS given its ‘epidemic of co-morbidities’ and a high 38% African American population (with a higher incidence of co-morbidities and risk of developing CRC) (Table 10) [5] surely need to be addressed in a systematic manner sooner than later [47].

Cost considerations also must be included in decision making, not only when making choices of screening tests of choice for individuals, but also for CRCS programs and for CRCS strategies in opportunistic encounters - even more so for MS. The most important fact is that the use of any screening modality is cost-effective (in addition to saving lives) than no screening [10,49]. Barzi et al. [48] utilized Markov plus Micro Simulation Screening Analysis (MISCAN) models to determine the comparative effectiveness of CRCS strategies. They used a validated natural history of CRC and 13 different screening strategies. The outcomes were measured in terms of discounted life years (years of future life lost) and the number of prevented CRC cases. Cost comparisons were measured using total cost, cost of screening, and cost of cancer care. Cost effectiveness was measured by Incremental Cost Effectiveness Ratios (ICERs) and incremental life years gained (LYG). This analysis showed colonoscopy, CT colonography, and flexible sigmoidoscopy as the most effective initial screening options in that order. Fecal DNA testing was more effective than other human hemoglobin detecting FOTs, but only by a small margin. Other important findings are listed in Table 11.

Rex et al.'s [6] findings were also consistent with findings listed in Table 11: (a) Colonoscopy was superior to other tests in most modeling analyses, and the traditional tests are more cost-effective than the newer modalities such as CT colonography, FIT-fecal DNA, capsule colonoscopy, and the Septin9 assay. If the newer tests improve compliance, then their cost-effectiveness may improve. However, these data are not yet available. Risk-stratified approaches to CRCS may improve cost-effectiveness; however, they still need more data to make evidence-based decisions. Risk-stratified approaches are likely to be extremely relevant to MS given the population's high-risk profile as detailed earlier and by Koutha et al. [5] in the parallel report by the authors of this paper. 

Direct visualization ‘invasive’ CRCS procedures - sigmoidoscopy and colonoscopy

The second group of tests available is based on direct visualization of the colon and rectum (Table 2). Colonoscopy procedure is considered the ‘gold standard’ for CRCS, at least in the USA among the asymptomatic population. The preference for colonoscopy is based on many considerations: (a) Ability to detect early, localized CRC amenable to surgical intervention and potential improved cures. (b) Ability to detect potential precancerous lesions and biopsy them; in many cases, resect them endoscopically or surgically resect them if sessile or too large to be removed endoscopically, thus preventing the adenomas from progressing to cancer. (c) These steps of detecting and removing precancerous lesions also identify a subset of the population that needs more intense surveillance, thus improving the overall secondary prevention of CRC. These steps improve overall survival and QOL outcomes in CRC [50-53].

Once the ability to safely perform flexible sigmoidoscopy was established, its potential in detecting cancerous and non-cancerous lesions was realized. Gangwani et al. [23] recently reviewed the historical development and innovations in colonoscopy. The upper gastrointestinal (GI) and sigmoidoscopies preceded the use of colonoscopy. The first (rigid) sigmoidoscopy, in fact, was performed as early as 1884. The use of flexible sigmoidoscopies (FS) started in earnest in the 1960s followed by the development of a wire loop snare-cautery device, thus facilitating removal of polyps in a single procedure [23]. The potential of FS as a CRCS test was recognized, and randomized phase III clinical trials showed the success of that approach. For example, Atkin et al. [24] reported the results of a randomized study of FS versus no FS with a median 11.2-year follow-up in 2010. This multicenter study was conducted in the UK among 14 centers. Outcomes were measured both by intention (FS)-to-treat (vs. no FS) as well as actual successful performance of FS (vs. no FS). The primary outcome measurement of CRC incidence in the former analysis decreased by 23% (hazard ratio 0·77, 95% CI 0·70-0·84) with the use of FS and mortality by 31% (0·69, 0·59-0·82). In the latter ‘per-protocol’ analysis, the CRC incidence among those screened with CRC was reduced by 33% (0·67, 0·60-0·76) and mortality by 43% (0·57, 0·45-0·72). More remarkably, the incidence of distal CRC was reduced by 50%. The numbers needed to be screened (NNS) to prevent one CRC diagnosis were 191; to prevent one death, it was 489. A 21-year update of the results in 2024 by Wooldrage et al. [25] showed sustained results seen at the 10-year mark. The Hazard Ratios (HR) for reduction in incidence were 0·76 favoring the FS group, and for death the HR was 0.75. The HR for distal colon incidence and mortality reductions were 0.59 and 0.55, respectively. The HR for the proximal colon incidence reduction and mortality were 0.98 and 1.0, thus showing the shortcoming of FS as a CRCS procedure in that only distal CRC outcomes were improved, and that is not a surprise since the FS procedure does not visualize the proximal colon.

The disadvantage of the lack of outcome benefits for the cancers in the proximal colon with the use of FS must be balanced with the requirements for less intense colon preparations, no or lesser need for sedation, extremely rare need for anesthesia and being a more cost-effective procedure compared to colonoscopy - FS costs range from $150 to $750, while colonoscopy costs range from $1,250 to over $4,000. The potential rare complications of bowel perforation and bleeding from both procedures are comparable.

Wang et al. [26] conducted a meta-analysis and systematic review on the influence of FS as a CRCS test. Their findings, reported in 2023, confirmed the results of 14-Center UK trial’s findings noted above. They identified six randomized clinical trials (RCT) plus one cohort study that met the criteria of their study requirements. There were 702,275 individuals from these seven studies who qualified for their analysis. A 26% relative risk (RR) reduction in CRC incidence (RR, 0.74; 95% CI, 0.66-0.84) and a 30% RR reduction in CRC mortality (RR, 0.70; 95% CI, 0.58-0.85) were noted. Many subgroups (men, women, distal site, stages III-IV, ages 55-59, and age over 60) were analyzed and found to have similar findings in incidence and mortality reduction with FS-based CRCS versus (a) no CRCS, (b) FIT-based CRCS and (c) ‘usual care’ [26].

Wilk and Niv [51] performed a meta-analysis and systematic review for different end-results outcomes (detection rates and complications) with the use of colonoscopy as a CRCS test among asymptomatic ‘average risk’ population and reported their finding in December 2024. Among 2,897,025 individuals screened, 99.6% were asymptomatic. Colonoscopy reached the cecum in 97-99% of the procedures. The detection rate for CRC was 0.5% (95% confidence interval [95%CI] 0.4-0.7%) and for advanced adenomas was 7.6% (95%CI 6.2-9.3%). The bowel perforation incidence was 0.022% and the bleeding incidence rate was 0.148%. In view of the lack of long-term (15 years) survival benefit results in RCTs comparing colonoscopy CRCS with non-screened populations, other evidence must be considered. Table 12 shows a compilation of such evidence.

**Table 12:** Level II Evidence Showing Outcome Benefits of Colonoscopy-Based Total Colon Visualization (TCV) Plus Interventions in Colorectal Cancer Screening (CRCS) Focused on Mortality Reduction Outcomes (Forest Plot) from reference [59] is shown in Appendix 2. This figure has been reproduced with permission. The table was developed independently by the authors from references [54-60].

S. No	TYPE OF STUDY AND ‘CASES’	COMPARATOR ‘CONTROL’ GROUP	OUTCOMES COMPARED & SALIENT FINDINGS	REFERENCE
1	Retrospective Population based Colonoscopy population identified from Manitoba’s provincial physicians' billing claims database from April 1, 1987, to September 30, 2007 (24,342 men and 30,461 women)	General population by standardized mortality ratios (SMRs)	Overall CRC mortality reduction by 29% (SMR, 0.71; 95% confidence interval [CI], 0.61-0.82] 47% reduction in mortality from distal CRC (SMR, 0.53; 95% CI, 0.42-0.67) No reduction in mortality from proximal CRC (SMR, 0.94; 95% CI, 0.77-1.17) Reduction in mortality from distal CRC remained significant for follow-up beyond 10 years (SMR, 0.53; 95% CI, 0.31-0.84).	Singh et al. [54]
2	Case-control study Surveillance, Epidemiology, and End Results (SEER)-Medicare data was used Cases (diagnosed with CRC between ages 70 to 89 years) were identified Dates: From January 1998 through December 2002 who died because of CRC by 2007 were considered the cases 9,458 cases: (3,963 lesions in the proximal colon [41.9%] 4,685 in distal colon [49.5%], and 810 unknown colon sites [8.6%])	Three matched controls without cancer for each case SEER-Medicare noncancer population was used Controls selected from those enrolled in Medicare Part A and B from age 65 years Controls were matched for sex, year of birth, race, and parent SEER registry From all the potential matches for each case, three randomly assigned controls were assigned to each case, 27,641 controls	Compared with controls, cases were less likely to have undergone colonoscopy (odds ratio [OR], 0.40; 95% CI, 0.37 to 0.43) For distal colon, the association was stronger (OR, 0.24; 95% CI, 0.21 to 0.27) than for the proximal colon (OR, 0.58; 95% CI, 0.53 to 0.64) for developing CRC A reduced risk of death from CRC was associated with colonoscopy This association was stronger for the distal CRC compared to the proximal colon cancer	Baxter et al. [55]
3	Retrospective secondary analysis of prospectively collected data [from NPS among 7 centers] ‘Case’ cohort can be considered ‘average risk’ although all of them were referred for a ‘diagnostic colonoscopy’ with the following initial positive findings: barium enema (27%), sigmoidoscopy (15%), FOBT (11%), or other tests (10%), symptoms directed TCV (32%) or a family history of CRC (5%) All underwent polypectomy	For CRC [incidence-based] mortality: SEER 9 data For all-cause mortality: National Center for Health Statistics (NCHS) Internal Control Population: Those who had resection for non-adenomatous polyps	Median follow-up = 15.8 years Cases: 12 deaths from CRC in (adenoma cohort) 1246 patients had died from any cause Controls: 25.4 expected deaths from CRC [NCHS data] Standardized incidence-based mortality ratio = 0.47 (95% CI, 0.26 to 0.80) That is, 53% reduction in mortality from CRC < 10 vs. > 10 years mortality comparison results were similar CRC Mortality rates were similar between ‘adenoma’ vs. non-adenoma populations All-cause mortality was lower in the ‘case’ (adenoma) cohort than in the ‘control’ general population [(matched by age, sex, race, and calendar year based on SEER9 data) standardized mortality ratio = 0.85 with a 95% CI, 0.81 to 0.90)]	Zauber et al. [56]
4	Retrospective secondary analysis of prospectively collected data Males: From Health Professionals Follow-up Study (51,529, 40 to 75 years of age at enrollment in 1986) Females: From e Nurses’ Health Study (121,700, 30 to 55 years of age at enrollment in 1976) Cases: Based on returned survey mail as consents - 88,902 total participants (31,736 men and 57,166 women) An incidence of 1815 CRC cases (714 men and 1101 women) during 22 years of follow-up 125 deaths from CRC 71 deaths from proximal colon cancers 37 deaths from distal colorectal cancers	Same male and female populations as in the ‘cases’ cohort, but had never undergone CRCS endoscopies 349 deaths from CRC 121 deaths from proximal colon cancers 195 deaths from distal Colo-rectal cancers	Lower mortality from CRC in the population with CRCS with sigmoidoscopy (multivariate hazard ratio, 0.59; 95% CI, 0.45 to 0.76) than among those who had never undergone CRCS endoscopy Lower mortality from CRC in the population with CRCS with colonoscopy (multivariate hazard ratio, 0.32; 95% CI, 0.24 to 0.45) than among those who had never undergone CRCS endoscopy Lower mortality with CRCS colonoscopy from both distal colorectal cancer (multivariate hazard ratio, 0.18; 95% CI, 0.10 to 0.31) and proximal colon cancer (multivariate hazard ratio, 0.47; 95% CI, 0.29 to 0.76) Lower mortality from distal CRC (multivariate hazard ratio, 0.31; 95% CI, 0.20 to 0.49) with Screening sigmoidoscopy Note: The other results of a decrease in the incidence of CRC and details of molecular analysis from this report are not shown here	Nishihara et al. [57]
5	Case-control study from the Veterans Healthcare System in the USA Cases: Veterans who died of CRC between January 1, 2002, and December 31, 2010 Veterans with CRC but who died of other causes were excluded Also excluded were those with a history of CRC or surgical resection of CRC prior to January 1, 2002, as well as those with Crohn’s disease, ulcerative colitis, and familial polyposis Cases were categorized as right-sided (cecum, ascending colon, hepatic flexure, transverse colon) cancer patients or left-sided (splenic flexure, descending colon, sigmoid colon, rectum) ones	Controls: Veterans with no diagnosis of CRC (prior to the index date) Also, they did not die of CRC prior to December 31, 2010. Case to control ratio: 1:4. STUDY DESIGN: National VA-Medicare administrative data were used to determine if colonoscopy was associated with decreased CRC mortality among predominantly male (99.3 % of all cases and controls) Veterans Also, if the outcomes differed by anatomic location (right vs. left colon) Controls were matched with cases by age (± 1 year), sex, and VA medical center, similar to cases Exclusion criteria were like the cases cohort. Controls had to be alive at the time of death of a matched case patient Electronic data sources for cases and controls: VA Central Cancer Registry (VACCR), VA Medical SAS datasets, Linked VA-Centers for CMS data, Department of Defense (DoD) Suicide Data Registry (SDR) and Pharmacy Benefits Management Services (PBM)	The indications for Colonoscopy were diagnostic in 68.7 % and 60.9 % and screening in 15.3 % and 21.3 %, and surveillance in 16.0 % and 17.8 %, for cases vs. controls, respectively (p = 0.0002) A total of 4,964 cases and 19,856 controls were identified; mean age (± SD) was 70.7 ± 10.0 years Cases had a higher Charleston Co-morbidity Score [thus this cohort is expected to have a higher mortality than the controls] The proportion of patients exposed to colonoscopy was 13.5 % for cases vs. 26.4 % for controls (p<0.0001) 668 cases and 5,250 controls exposed to colonoscopy Cases were less likely than controls to have undergone any colonoscopy (adjusted OR=0.39; 95% CI, 0.35-0.43) Colonoscopy was associated with reduced odds for left-sided cancer (aOR=0.28; 95% CI, 0.24-0.38) as well as right sided cancer (aOR=0.54; 95% CI, 0.47-0.63) Screening Colonoscopy Cohort: Adjusted ORs were 0.30 (95% CI, 0.24 -0.38) overall, 0.20 (95% CI, 0.14 0.27) for left-sided cancer, and 0.48 (95% CI, 0.35-0.66) for right-sided cancer CRC mortality reduction among Veterans associated with Colonoscopy was 61 % overall Mortality reduction was seen both for left-sided and right sided CRC Association was weaker for right-sided cancer (46 % versus 72 % for the left sided CRC) [that is reduction in right-sided colon cancer deaths was less than that for left-sided cancers] Similar results were noted among the Screening colonoscopy subgroup cohort	Kahi et al. [58]
6	Nested case–control study Cases and controls selected from Kaiser Permanente (KP) Northern California (KPNC) and Southern California (KPSC) KP is a nonprofit health plan and hospital system focused on community-based health care It is an integrated prepaid multispecialty medical group Patient and health care subjects represent a racially, ethnically, and socioeconomically diverse population Cases: 55–90 years old men and women enrolled in KP system between 1 January 2006 and 31 December 2012 who died of CRC All had ≥5 years of enrolment prior to their reference date [the diagnosis date] At KPSC, cases were accrued from 2011 and 2012 calendar years	Individually matched control for each case using an incidence-density matching approach This approach helped reduce socioeconomic variation between cases and controls and thus minimized selection biases Matching was done based on the reference date on birth year (±1 year), sex, the duration of health plan enrolment prior to diagnosis (±1 year), and the geographical region All cases and controls were ‘average risk’ cohorts High-risk population with history of IBD, CRC in ≥1 first-degree relatives before age 50, or ≥2 first degree or second-degree relatives at any age, or those with familial CRC syndromes, or those who had GI cancer or colectomy before the reference date was all excluded	1747cases and 3460 matched controls About 50% were females The majority was non-Hispanic whites and had≥10years of health plan enrolment and without any significant comorbidities Compared to controls who did not receive CRCP, those who underwent CRCP had a 67% lower risk of dying from CRC (aOR=0.33; CI0.21to 0.52) There were no differences in the mortality risk reduction between right versus left colon cases (p value=0.51) A figure illustrating the results is reproduced below with permission [shows the mortality reduction for the whole group as well as ‘sub-groups’] In the figure: *Patients excluded from the primary analysis (underwent both colonoscopy and sigmoidoscopy) and were coded as screening sigmoidoscopy. **Patients excluded from the primary analysis (underwent both colonoscopy and sigmoidoscopy) and were coded as screening colonoscopy.	Doubeni et al. [59]. Note: Figure included for this reference in this table is reproduced with permission.
7	Case Control Study Cases (5128) and controls (20,512) were Utah residents Age Range: 54 to 90 years CRC diagnosis was made from 2000 through 2010. The unique linkage between the Utah Population Database and Cancer Registry was used Advantages of this linkage: High-quality CRC diagnosis confirmation as well as colonoscopy procedures determination in the whole state of Utah Good family history intake was possible, for example, familial CRC history without obtaining them using secondary sources-bias [ascertainment, referral or recall] Covariates used and adjusted in statistical models: (a) Family history of CRC, alcohol consumption or cigarette smoking [known risk factors for CRC] (b) Family history of CRC in a first degree relative was determined from the unique linkage of genealogical and cancer registry records with the UPDB (Utah Population Database); (c) Affiliation with the Church of Jesus Christ of Latter-day Saints (or Mormons) – whose members follow principles of proscriptions against alcohol consumption and cigarette smoking [used in the regression models to control for the variable]	Controls: Age and sex matched persons with no history of CRC Matched for each case Receipt of colonoscopy 6 months to 10 y before the reference date for each case and control through administrative claims data Colonoscopy exposure was compared by using conditional logistic regression The significance of this study: Statewide; no recall and other biases; not limited to ‘special populations’ such as: Medicare only population; Veterans only population; HMO [Health Maintenance Organization] only population. 741 of cases (14%) and 5,715 of controls (28%) received a colonoscopy. Exclusions: A diagnosis of CRC, history of Crohn’s disease (ICD-9 code 555), ulcerative colitis (ICD-9 code 556) or molecular genetic diagnosis (confirmed APC) adenomatous polyposis (FAP) mutation)	End Points: New-onset CRC and death from CRC Secondary Endpoints: Effectiveness of colonoscopy in different genders, age groups, and cancer stages. Exposure to colonoscopy reduced the odds for a diagnosis of CRC OR: 0.41 for any CRC (95% confidence interval [CI], 0.38–0.44) OR: 0.58 for proximal colon cancer (95% CI, 0.51–0.65) OR: 0.29 for distal colon or rectal cancer (95% CI, 0.25–0.33). Exposure to colonoscopy reduced the odds for death from CRC OR: Decreased odds of death from CRC, 0.33 (95% CI, 0.28–0.39), OR: proximal colon 0.43 (95% CI, 0.34–0.55) OR: Distal colon or rectum 0.23 (95% CI, 0.18–0.30) These results were consistent among sexes, age groups, and cancer stages. All-Cause Mortality was also reduced with Colonoscopy: association of a reduced odds of death from all causes; both for proximal colon cancer and distal CRC; the effect stronger for distal CRC (OR 0.27, 95% CI: 0.22–0.33) compared to proximal colon cancer (OR 0.54, 95% CI: 0.45–0.65)	Samadder et al. [60]

Of the seven studies detailed in Table 12, many were limited to special populations such as Medicare-only (age restricted to above 65 years) subjects, veterans only (predominantly males) group, or participants of a Health Maintenance Organization (HMO). Those restrictions can lead to selection biases, and the applicability of the findings to a ‘general, community-clinical-practice-confined-populations’ is not known. It should be noted that none of the seven studies in Table 12 included a sizeable percentage of the African American population. Nevertheless, direct visualization methods of colonoscopy or FS do offer benefits with some disadvantages noted earlier, and with a patient and/or family-focused personalized approach, the benefits can be enhanced and the harms decreased.

Non-invasive direct visualization (NIDV) CRC procedures - CT colonography (CTC) and colon capsule endoscopy (CCE)

NIDV procedures - CTC and CCE - have many similarities and some differences and for that reason, they are discussed together. Here are the commonalities: (a) Can visualize the whole colon and rectum. (b) Non-invasive procedures. (c) Less expensive than a colonoscopy or FS. (d) Less interruptive work schedules than colonoscopy or FS, since there is no need for sedation or anesthesia, and hence the associated recovery time. (e) Both require bowel preparations. And here are the differences: (a) CTC uses radiation, but the dose is negligible according to published medical evidence. CCE does not use ionizing radiation, although the radiation dose from CTC is less than other diagnostic evaluating CT scans of the abdomen and pelvis CCE captures the images of the bowel using high-fidelity cameras and sends the images for capture to a recorder. (b) CCE technique requires swallowing of a miniature camera to catch the images of the bowel system. Occasionally, the capsule can get stuck in the bowels. (c) The only risk with CTC is colon perforation due to the insufflation of the colon with air or CO2, but that risk is less than .01%. For CTC oral contrast increases specificity, but CCE does not require them [61].

NIDV review will be confined to the most recent, important, relatively large-scale studies in view of the length constraints of this paper. Turvill et al. [62] recently reported results of a large, population-based, observational diagnostic accuracy study of CCE and compared the results to colonoscopy and CTC. The study was conducted by the NHS in the UK during the COVID-19 pandemic. Because of the limitations of the ability to perform colonoscopy and/or CTE during COVID-19 times due to the concerns of potential further spread of COVID-19 from procedures such as colonoscopy of FS, CCE was offered as an alternative to ‘intermediate risk’ patients in the NHS. Patients with FIT results of ≤ 100 μg Hb/g feces or those with potential contraindications for colonoscopy or CTC were offered three choices to choose: colonoscopy, CTC, or CCE. Patients with potentially higher risks of CRC (for example, FIT results of > 100 μg Hb/g feces) were offered CTC or colonoscopy. These clinical decision tree recommendations were arrived at with the help of an Expert Advisory Group (EAG). The resultant population undergoing all three procedures - colonoscopy, CTC, and CCE - serves as a relatively unbiased CRCS eligible group. This relatively high-risk group for CRC choosing one of the three CRCS tests during a concurrent time provides a unique opportunity to compare the relative efficacies of them as CRCS alternatives after a positive FIT, in a ‘Two-Step’ model (Table 11). Since most individuals at risk for CRC choose FIT against colonoscopy as the first CRCS test of choice, this study is an important evidential step assessing these three CRCS alternatives, although not a randomized study with 10,369 patients, 1900 of whom had FIT < 10 μg Hb/g feces. The results showed a CCE as a remarkable noninvasive alternative that can avoid colonoscopy under certain carefully selected circumstances: (a) The results are for 47% (4878 patients) of 10,369 who chose CCE; 48.5% (5025 patients) who chose colonoscopy; and 4.5% (466) who chose CTC. (b) 98.4% of CCE patients tolerated the procedure well and performed safely. (c) CCE was able to identify every matched mass lesion among those pathologically diagnosed with a CRC (when CCE was completed in adequately bowel-prepared patients). (d) CCE detected more polyps ≥ 10 mm as well as 6-9 mm ones compared to colonoscopy or CCT, and CCE’s per-patient sensitivity was 97% for those lesions. (e) However, the CCE’s completion rate was only 74%. (f) The CCE population’s comorbidities are reflected in a 74% bowel preparation adequacy rate versus 88% for colonoscopy or CTC populations. The authors concluded that “CCE is a safe diagnostic test of CRC and unlikely to miss significant disease” and can… “act as a filter test”. They further said that CCE could “appropriately” help in “informing the onward management of the patient in a resource-constrained healthcare system.” Given the resource constraints in the state of MS, CCE may have a sizeable role to play in the state. In terms of complications, the inability to swallow the capsule, vomiting and suspected retention were the most common ones - 0.3%. 0.4% and 0.7%, respectively. Three emergency laparotomies were performed on patients after CCE (0.06%). There was one death among 4878 procedures (0.02%) as well as two perforations (0.04%). There were 64 (1.3%) technical failures with CCE (failure of the camera to capture right‐sided colonic images was the main issue).

**Figure 1:**
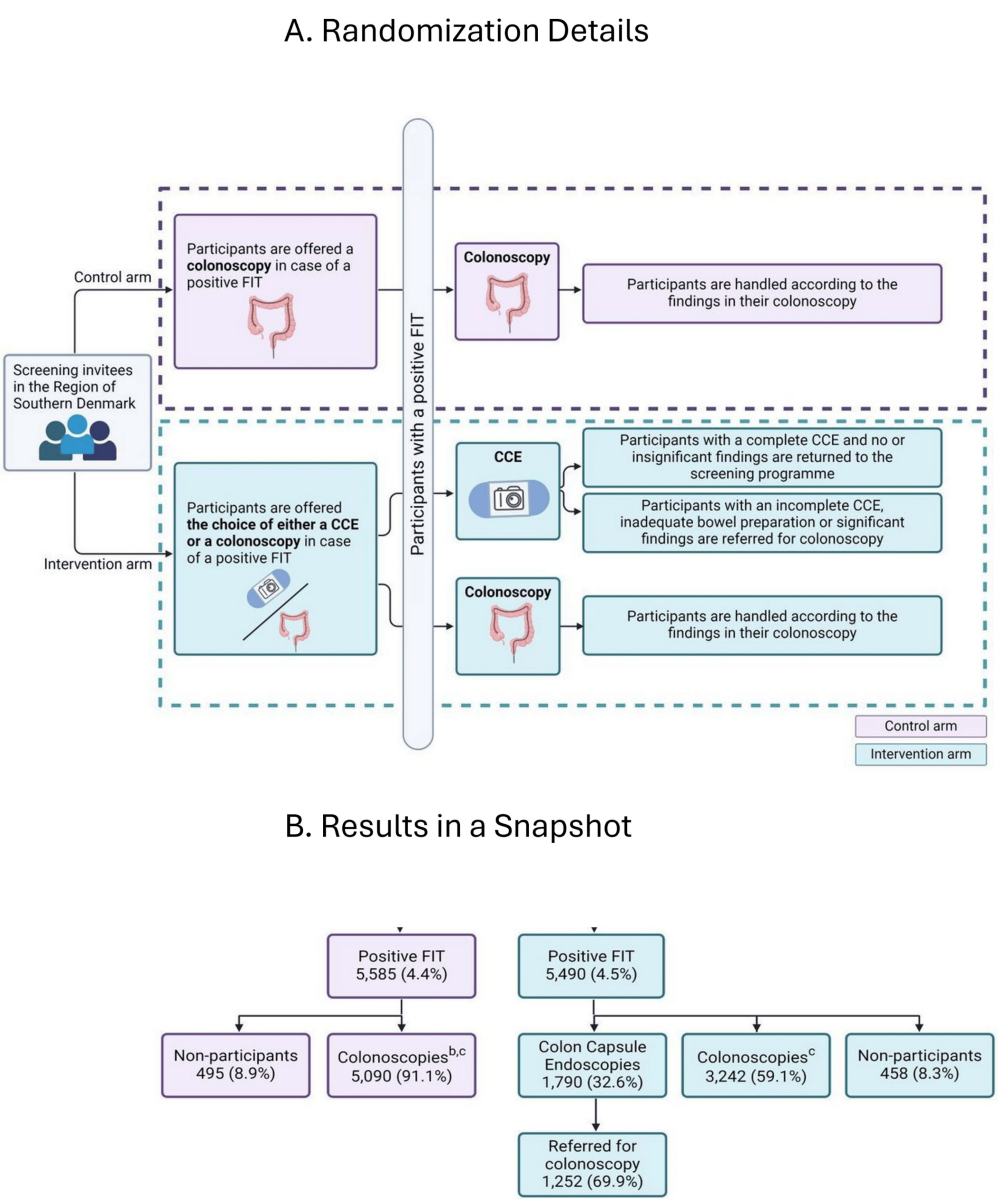
Population-Wide, Cluster Randomization Study from Southern Denmark A: Randomization Details; B: Results in a Snapshot “Study design: individuals are invited to the trial before the fecal immunochemical test (FIT). Data was collected on FIT-positive individuals only. They are allocated to the two treatment groups in alternating weeks”.  Figure has been reproduced with permission from the source [63] CCE = Colon Capsule Endoscopy

Baatrup et al. reported the results of a randomized study by the ‘CareForColon2015 Study Group’ from Southern Denmark in April 2025 [63], using cluster randomization method. This was an intention-to-treat, multicenter, open-label, parallel group-controlled population-wide study covering about 1.3 million people. All four hospitals in the Southern Denmark’s Healthcare Region participated. The study design is described in relative detail since similar designs in DS states can improve compliance to CRCS and uptake. Figure 1A, 1B (reproduced with permission from Baatrup et al. [63]) show the alternative weekly randomized groups in the control and intervention arms and a brief ‘snapshot’ of the results, respectively. Among 473,684 invitations that were sent equally divided among the two arms, 63.5% and 61.7% returned the FIT kit. The returning of the FIT kit was considered ‘the consent’ for the study; 4.4% and 4.5% of control and intervention groups had a positive FIT (Figure 1B). In the control arm, 91% accepted the colonoscopy and 9% declined. In the intervention arm, 45.8% preferred CCE, and 11.4% preferred colonoscopy. A high 42.8% did not respond to either of the two options between CCE versus colonoscopy and were assigned to undergo colonoscopy (Figure 1B). Adequate bowel preparation and complete transit were accomplished in 69% of CCE participants. Surprisingly, 70% of CCE participants ended up requiring colonoscopy for either a positive finding in the CCE or due to inadequate CCE study. The percentage of advanced neoplasia detection was 0.67% and 0.64% in the control (colonoscopy only) arm versus the intervention of CCE choice arm - not different. Although the authors concluded that CCE resulted in a very high ‘secondary’ colonoscopy rate with this trial design and was not recommended, there are other extremely useful lessons that can be learnt from this study. No bowel perforations, deaths or laparotomy interventions as complications of CCE were reported. The concerns of a need for a high follow-up endoscopy rate after CCE are also reflected in another recent (May 2025) CCE systematic review and meta-analysis report by Lei et al. [64]. Among 2850 participants from 19 studies, these European authors found a follow-up endoscopy rates of 0.10-0.15 after upfront-colonoscopy, 0.25 for CTC and 0.42 (95% CI (0.34 to 0.50) for CCE. Both Baatrup et al. and Lei et al. advocate more research to improve the importance of complete and satisfactory bowel preparations, capsule transit and other potential technical advances (such as the use of artificial intelligence, AI) to improve CCE’s utilization as a viable option after a positive FIT test.

Although the initial patent for CTC [65,66] was granted as early as 1994, it took almost 20 more years for CTC to become a reality in clinical practice, especially CRCS. As shown in Table 1, CTC is one of the recommended NIDV procedures for CRCS under specific circumstances. CTC is used more in other Organization for Economic Cooperation and Development (OECD) countries than in the USA. There is a certain nuanced use of CTC (and CCE) in some of the OECD nations, such as Canada and the United Kingdom (UK), that can be followed in certain geographical areas within the USA. Canada and the UK have universal health care programs; this leads to a need for greater utilization reviews and a nuanced approach to using highly expensive (technical) procedures [67,68]. One of the reasons for this is the lack of some of the infrastructure availability widely, as well as more centralized specialized care centers. Due to these factors, there are some similarities in terms of access issues between these countries and the DS. Hence, two recent studies - one from Canada and the other from the UK - will be reviewed next.

**Figure 2:**
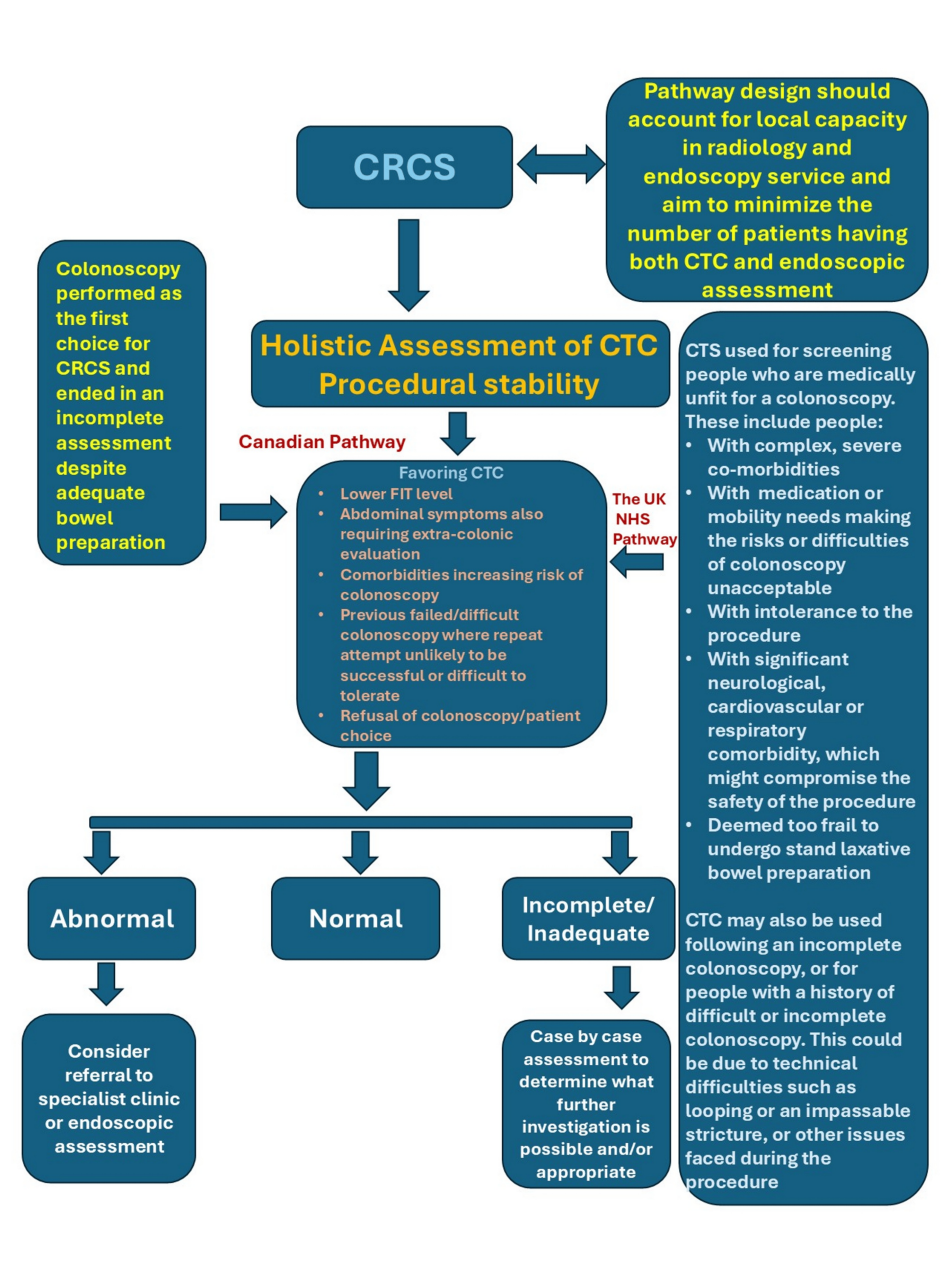
Appropriate Use of CT Colonography (CTC) in Colorectal Cancer Screening (CRCS) Based on Canadian and the United Kingdom National Health Care Practice Guidelines Synthesized by authors from the discussion above from [69,70]. "Since these concepts were developed in two countries with fewer resources that the USA with national health systems conscious of the need for efficiency and cost-benefit' considerations, these ideas can be useful in resource-constrained states like Mississippi"

The Canadian Association of Radiologists (CAR) published their Practice Guidelines (PG) for CTC in 2024 [69]. Similarly, PG was updated for the use of CTC within the NHS recently (October 2024) [70]. Based on these two guidelines, Figure 2 is designed to detail the appropriate use of CTC. In these two nations, CTC is accepted only for those who are not safe to undergo colonoscopy (co-morbidities risking high chances of complications with sedation/anesthesia) or an incomplete colonoscopy despite adequate bowel preparation. Other reasons are detailed in Figure 2.

Based on these recent data reviewed on CTC and CCE, the following conclusions may be drawn: (a) Both procedures do have advantages due to their noninvasive nature, and if further improvements can be made with technical advances such as AI, they may become a first-line choice to screen FIT-positive patients to further decrease the colonoscopy rates. (b) CCE may have further advantages than CTC due to a lack of the risk of ionizing radiation use as well as potential use at home without the need of a trip to a center with CTC capabilities. (c) a and b listed above will become practical only if the subsequent colonoscopy and/or FS rates can be significantly reduced with improved sensitivity and specificity of CCE and/or CTC. (d) In rural geographies in the US as well as in other nations, CTC and/or CCE can have beneficial applications, especially if further advances happen as outlined in a-c above.

Blood tests for CRCS

**Figure 3:**
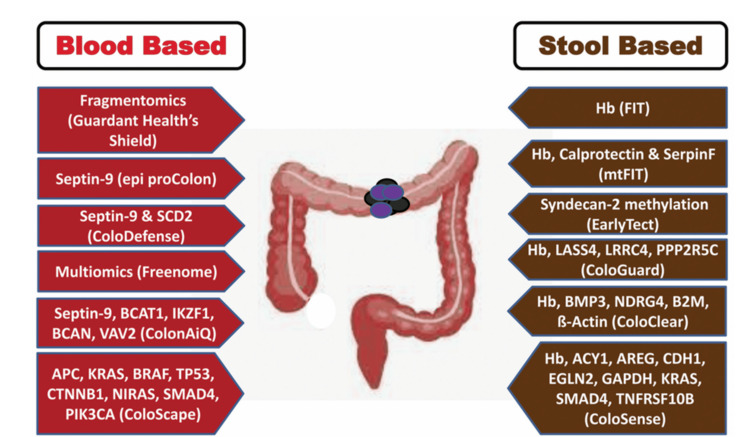
Blood-Based and Stool-Based Colorectal Cancer Screening (CRCS) Tests: Currently Available As Well as Soon-To-Be-Available Candidates Reproduced with permission from [73]. This figure content is free to use when original source is cited. FIT = fecal immunochemical test

Blood tests have the advantage of easier acceptance due to the ability to be performed at a provider’s office/clinic (or even at home) than the other tests listed above as well as the familiarity of blood draw that is so routine and familiar to almost any adult who is at risk for CRC. The lack of a need for extensive infrastructures can also make them more cost-effective [30,71]. However, as noted in NCI’s cancer.gov detailing the screening tests to detect CRC and colonic polyps, blood tests for CRCS are yet to be incorporated into clinical guidelines [72]. Two blood tests are FDA-approved in the USA as of now. The first test is for a molecular biomarker, methylated SEPT9. This qualitative PCR assay to detect methylated Septin9 DNA shed by CRC cells into the bloodstream was approved in 2016 [28]. The test is called Epi proColon 2.0 and is approved to be used to screen adults 50 years or older at average risk for CRC. The at-risk’ population is those who have been offered and have a history of not completing CRCS using a stool test or a direct visualization test. The second test, named ‘Shield’, analyzes plasma DNA for the presence of harmful gene variants again is approved for average risk adults aged 45 and older. The ‘Shield’ has a specificity of 90% and a sensitivity of 84%. Agarwal et al. [73] conducted a survey among health care professionals and their parents in India. This recent study was focused on the uptake rate of colonoscopy among highly educated and knowledgeable people. Among 2,199 adults (733 health care professionals and the rest were their parents) only 7.13% underwent CRCS-colonoscopy, knowing that they knew the benefits of CRCS. Agarwal et al. [73] point out the invasive nature of colonoscopy, the rigors of bowel preparations, operator dependency on the sensitivity and specificity rates, as well as a small, yet potentially life-threatening risk of serious complications associated with colonoscopy as the barriers. In the ‘real world situation’, they point out, a two-step process can yield better uptake rates of CRCS. The first step is easy to perform, relatively infrastructure-free, and potentially universally accepted blood test-based CRCS. If found positive, Agarwal et al. [73] argue that the uptake for CRCS-colonoscopy will be higher. This hypothesis needs to be demonstrated in practice using phase I / II clinical trials among average-risk and high-risk populations, especially in communities and geographies with limited immediate easy access to colonoscopy. One also has to include the cost-effectiveness of such an approach, as there is an ongoing debate in the literature on the merits and demerits of cost-effectiveness [32,74]. Suffice to say that the use of blood-based CRCS using so-called liquid biopsies is an area of extremely active research and development [30,75]. Great breakthroughs are surely expected and likely to play a greater role in rural and disadvantaged communities and geographies such as MS. In a commentary on the Clinical Practice Update by Shaukat et al. [75] published in April 2025 the nuances associated with the use of blood-based tests in CRCS are well articulated and again emphasize the importance of population-based clinical trials in underserved populations who are the ones who are in real need of such innovations [76]. The status of the globally available blood tests in CRCS is depicted in Figure 3.

Discussion

CRCS is a strategy that has a great track record of saving lives with a sufficient percentage of compliance and resultant adequate uptake. This happens with the detection of precancerous lesions and appropriate removal of them, thus preventing progression to malignant tumors, or by diagnosing CRC at earlier stages. These dynamics involved with CRCS lead to improved survival outcomes, demonstrated mainly in countries with advanced economies and adequate infrastructure, especially colonoscopy. The data reviewed here shows that these advantages of CRCS must be further refined to be effective among rural, disadvantaged, and infrastructure-deficient geographies and less educated and less informed communities and populations. One-size-fits-all approaches cannot and will not yield desirable as well as cost-effective outcomes. As reviewed in detail earlier in this article, choosing the right CRCS test(s) is an extremely complicated and complex task. The stakeholders are too many - at-risk populations, care providers, hospital administrators, health policy makers, politicians, governmental decision makers, national and international agencies, drug, vaccine, and equipment making for-profit entities - the list can go on. The goals of all these stakeholders must align with one purpose - a ‘at-risk-population focused strategy’ - to improve CRC outcomes by designing the ‘right CRCS strategy for the right population at the right time’. The innovations and technological improvements must be customized to each human individual at risk.

**Figure 4:**
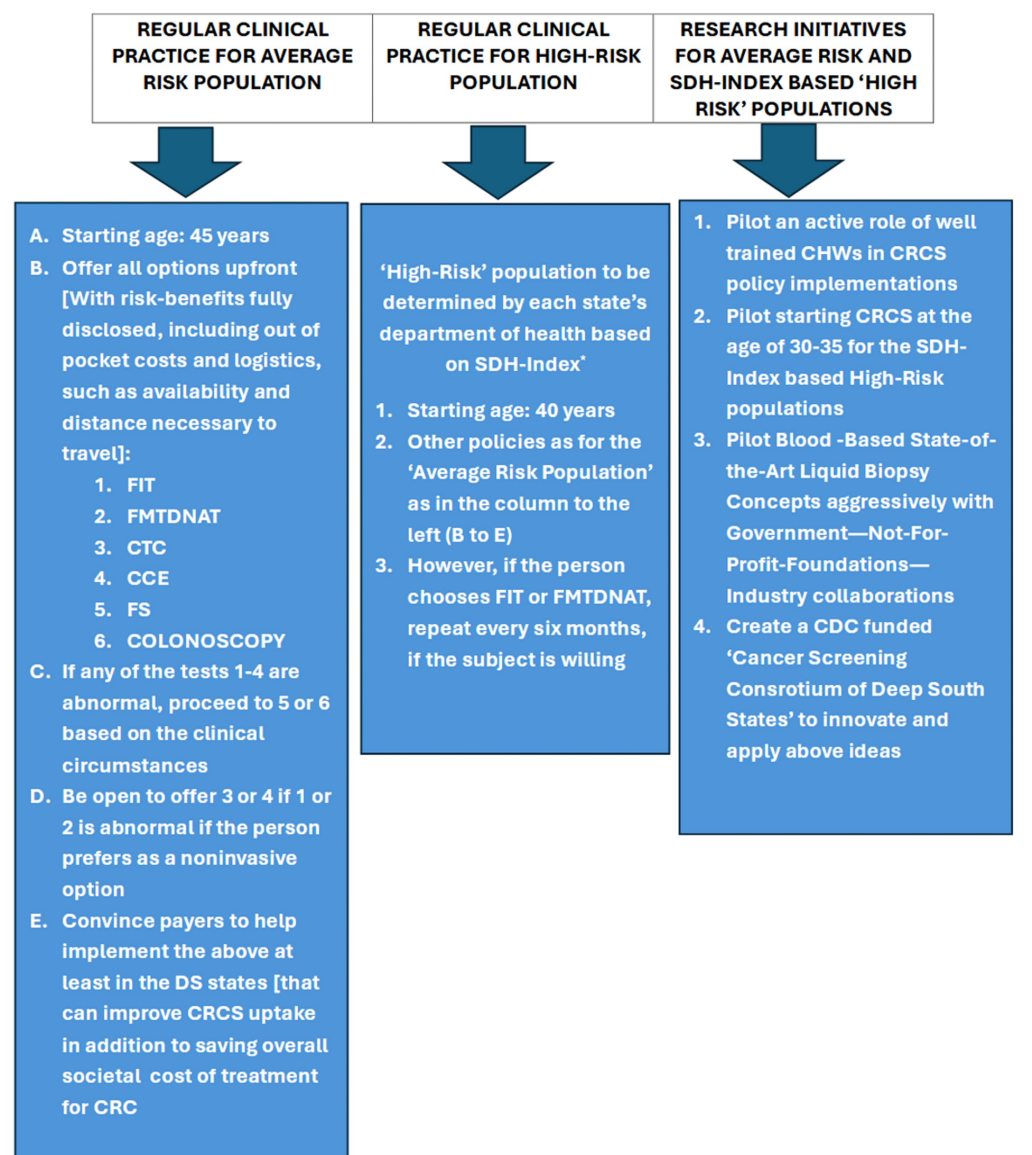
A Conceptual Construct for Practice and Research for Quick Implementation Figure developed by authors independently CHWs = community health workers, CRCS = Colorectal cancer screening, FIT = fecal immunochemical test, FMTDNAT = fecal multi-target DNA test, CTC = CT colonoscopy, CCE = colon capsule endoscopy, FS = flexible sigmoidoscopies, DS = Deep South

This paper and the accompanying parallel paper [5] propose unique interdisciplinary data-driven approaches to CRCS. For example, in the parallel paper by Koutha et al. [5] the potential role of CHWs is discussed and emphasized. This proposal of a role for CHWs is supported by a recent clustered randomized study conducted in 28 rural clinic units in rural Oregon [77]. These clinic sites were all affiliated with three Medicaid health plans in the state of Oregon in the USA - thus representing underserved populations. Fourteen clinics followed regular, standard practices of CRCS. The second group of 14 facilities had stepwise interventions - consisting of mailed FIT outreach and then patient navigation. Practice facilitation methods and training collaborative learning as well as patient tracking tools were used. Those clinics randomized to interventions showed improved CRCS primary and secondary outcome goals than the standard practice arm. In June 2025, Chia et al. [78] introduced the idea of using FIT at an earlier age compared to focusing on colonoscopy. FIT at 40 (FIT 40) or 45 (FIT 45) years led to more life-long colonoscopies and FIT 40/45 also reduced the incidence of CRC and deaths from CRC. FIT 40/45 also showed for QALY gained. In an editorial, Richard Wender (2025) proposes “Start(ing) Younger, Offer(ing) Choice(s), (and) Find(ing) Advanced Precancerous Lesions (early)” to decrease the incidence of CRS and decreasing the mortality to achieve “Maximum Benefit from” CRCS [79]. These ideas, proposals and concepts are similar to the ones being put forward in this communication (Figures 2, 4).

Based on the review of the recent English language’s relevant research literature by an interdisciplinary team that included an oncologist, an epidemiologist, a cancer control policy expert, an experienced leader of community healthcare workers, a community health and population medicine academician, a radiologist with expertise on CRCS nuances and an internist-radiologist - a conceptual construct for practice and research is proposed (Figure 4).

The proposal for the average risk population follows the age recommendations of professional societies in Table 1 and the currently available options expanded in Tables 2-12 and Figures 1-3. The emphasis is on starting the CRCS at a younger age (of 45 years) and offering as many available approved options as possible to the person at risk and available community resources to match the acceptability of the person choosing it. The proposal defines the high-risk population in a unique way - while not excluding those at high risk for CRS based on genetic and familial history considerations - in that it includes high-risk persons also based on SDH-index [5]. For the ‘high-risk’ group, the proposal is also to start at an age of 40 years based on the available data reviewed earlier. For future research piloting of new ideas in the DS region of the USA is outlined that would require appropriate Institutional Review Board approvals and funding by federal and local governments as well as other not-for-profit foundations and for-profit corporations.

Although the paper focuses on one of the DS states in the USA - Mississippi - the success in MS and other DS states can be appropriately modified and successfully implemented among other similar resource-lean communities, geographies, and countries. In fact, the above approaches can make CRCS more effective not only in terms of saving more lives but also being more cost-effective and improving quality of life even among resource-rich environments [78,79].

Shortcomings

As noted under ‘Methodology’, this investigation used a different methodology of ‘stepwise’ interdisciplinary research than the traditional methodology of ‘PRISMA-like’ systematic review/meta-analysis. This methodology can be considered a major shortcoming. However, in interdisciplinary research, the progress of the outcomes that would be yielded by researching of the peer-reviewed literature cannot be predicted and hence the additional disciplines that need to be consulted and shared the original approach and subsequent hypotheses-generation for further research. The pros and cons of this approach are beyond the scope of this paper.

## Conclusions

In this report and a review published recently the CRCS options are reviewed with a lens that focuses on the issues relevant to resource lean and rural communities in the state of MS - although these data can be applied to many other DS states and regions within the USA and countries in GS. The paper can serve as a ‘spark’ that can stimulate further debate, hypothesis generation, new clinical and epidemiological studies, policy initiatives and new funding legislations. Ultimately the value of this paper will be measured not necessarily by the citation indexes, download-statistics or other metrics but rather by the improved CRCS uptake and lives saved in MS, DS and GS in the years to come.

## Figures and Tables

**Figure 5:**
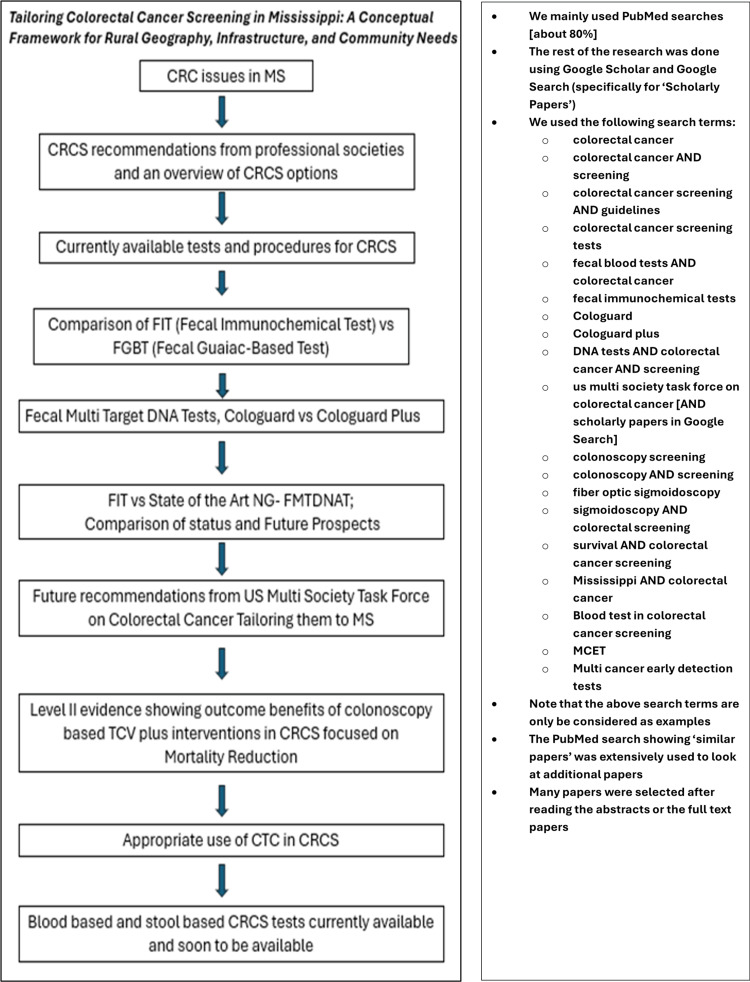
An outline of Search Methodology

**Figure 6:**
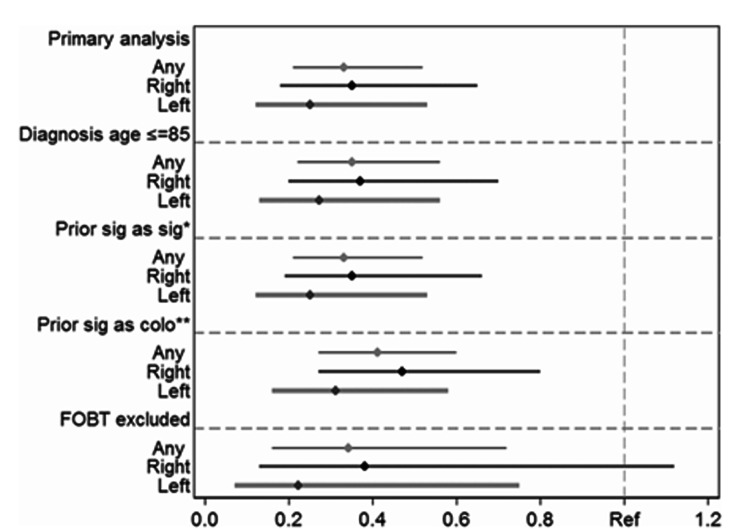
Forest Plot for the Case Control Study discussed in Table 12 Image reproduced from the cited source [59] with permissions

## Appendices

Appendix 1 

Appendix 2  
